# Supplementary material for: Infectious disease surveillance in U.S. jails: Findings from a national survey
Source: PLoS One. 2022 Aug 25;17(8):e0272374. doi: 10.1371/journal.pone.0272374 (PMC9409583; doi:10.1371/journal.pone.0272374)
Supplement: S1 Table — (DOCX) [file pone.0272374.s001.docx]

| **S2 Table.** Prevalence of jurisdiction size. | | | |
| --- | --- | --- | --- |
| Jurisdiction Size | BJS  (n = 2,463) | Responders Unweighted  (n = 371) | Responders Weighted, (se)  (n = 371) |
| Fewer than 50 inmates | 34.7% | 20.4% | 35.1% (0.029) |
| 50-99 inmates | 18.8% | 16.7% | 18.3% (0.021) |
| 100-249 inmates | 22.5% | 23.4% | 22.2% (0.022) |
| 250-499 inmates | 10.9% | 13.7% | 10.8% (0.015) |
| 500-999 inmates | 7.0% | 10.8% | 7.2% (0.011) |
| 1,000 or more inmates | 6.3% | 15.1% | 6.4% (0.009) |

Note: BJS uses jurisdiction size to create sampling weights for the Annual Survey of Jails. This is also stratified by whether the jail housed a juvenile at the time of the survey. Given the small study sample size and the problem of zero cell counts, only jurisdiction size was used for the current study. First, the inverse probability of being an AJA member (versus non-AJA member) was estimated based on jurisdiction size. Second, the inverse probability of responding to the survey (versus not responding) was estimated based on jurisdiction size. These two inverse probabilities were then multiplied for responding jails to create sample weights. The unweighted prevalence rates indicate that the study sample is underrepresented by small jails (e.g., jurisdiction size of fewer than 50 inmates) and overrepresented by larger jails (e.g., jurisdiction size of 1,000 or more inmates). The estimated prevalence rates using sample weights shows that the study sample can be successfully adjusted to closely approximate the national population of jails with regards to jurisdiction size.
