# Supplementary material for: Infectious disease surveillance in U.S. jails: Findings from a national survey
Source: PLoS One. 2022 Aug 25;17(8):e0272374. doi: 10.1371/journal.pone.0272374 (PMC9409583; doi:10.1371/journal.pone.0272374)
Supplement: S1 File — (PDF) [file pone.0272374.s002.pdf]

# National Survey of Health Care in U.S. Jails

The University of Miami (UM) and the American Jail Association (AJA) are conducting this survey. The survey has 5 parts including 23 questions. The questions ask about the health care practices and policies of your jail system including all of its facilities. The answers are not anonymous. However, findings in reports and publications will only be presented in aggregate form so that individual jails cannot be identified.

If you have any questions about completing the survey, please contact the UM Jail Study research team by email (jailstudy@miami.edu) or phone (305-284-6154).

If you have any additional questions or concerns, please contact the study principal investigator (Dr. Kathryn Nowotny) by email (kathryn.nowotny@miami.edu).

**<u>Survey Instructions</u>**
There are multiple options for completing this survey.

**By Postal Mail**
The survey can be completed and returned by postal mail to this address:

University of Miami Jail Study
5202 University Dr., Merrick Bldg, Rm 120
Coral Gables, FL 33146

For your convenience, we have included a preaddressed stamped return envelope.

**By Email**
The survey can be completed, scanned, and emailed to jailstudy@miami.edu.

**Online**
The survey is also available online through Qualtrics. If you would like a link to complete the survey online, please email this request to jailstudy@miami.edu.

## Pre-Survey Information

What STATE is your jail system located in? ______________________

What COUNTY is your jail system located in? ______________________

What is the NAME of your jail system? ______________________

## PART 1: Medical Intake

**QUESTION 1: Medical Intake**

Questions 2-6 are about your medical intake. For the purposes of these questions, medical intakes are the screening questions and activities conducted by health or custody staff after admission, before assigning someone to a housing unit.

Medical intake does NOT include: screening you do at the front door, while the arresting officer is still present, to decide whether you will accept or divert the arrestee; a more in depth evaluation that is done by a nurse or practitioner, typically called the 14-day health assessment.

Does your jail conduct any kind of medical intake? [circle one]

A. Yes

B. No

If "**yes**", continue to **QUESTION 2**.

If "**no**", skip to **PART 2: Non-Urgent Episodic Care.**

**QUESTION 2: Infectious Disease Screening**

Does your jail system screen inmates for the following infectious diseases during the medical intake?

| **INSTRUCTIONS:** Check all that apply (at least one response per row). | | | | | | |
|---|---|---|---|---|---|---|
| Infectious Diseases | Yes, ALL Inmates | | Yes, SOME Inmates | | No | Don't Know |
| | Self-reported by inmate | Tested with laboratory results | Self-reported by inmate | Tested with laboratory results | | |
| a) Hepatitis A | ☐ | ☐ | ☐ | ☐ | ☐ | ☐ |
| b) Hepatitis B | ☐ | ☐ | ☐ | ☐ | ☐ | ☐ |
| c) Hepatitis C | ☐ | ☐ | ☐ | ☐ | ☐ | ☐ |
| d) Gonorrhea | ☐ | ☐ | ☐ | ☐ | ☐ | ☐ |
| e) Chlamydia | ☐ | ☐ | ☐ | ☐ | ☐ | ☐ |
| f) Syphilis | ☐ | ☐ | ☐ | ☐ | ☐ | ☐ |
| g) Tuberculosis (TB) | ☐ | ☐ | ☐ | ☐ | ☐ | ☐ |
| h) HIV/AIDS | ☐ | ☐ | ☐ | ☐ | ☐ | ☐ |

If "some" inmates, please explain (e.g., only test upon request, only to confirm self-report, etc.). [write-in response]

**QUESTION 3: Physical Health Screening**

Does your jail system screen inmates for the following physical health conditions during the medical intake?

| **INSTRUCTIONS:** Check all that apply (at least one response per row). | | | | | | |
|---|---|---|---|---|---|---|
| Health Concerns | Yes, ALL Inmates | | Yes, SOME Inmates | | No | Don't Know |
| | Self-reported by inmate | Tested with laboratory or clinical results | Self-reported by inmate | Tested with laboratory or clinical results | | |
| a) Pregnancy (females only) | ☐ | ☐ | ☐ | ☐ | ☐ | ☐ |
| b) Elevated lipids/ cholesterol | ☐ | ☐ | ☐ | ☐ | ☐ | ☐ |
| c) High blood pressure | ☐ | ☐ | ☐ | ☐ | ☐ | ☐ |
| d) Diabetes | ☐ | ☐ | ☐ | ☐ | ☐ | ☐ |

If "some" inmates, please explain (e.g., only test upon request, only to confirm self-report, etc.). [write-in response]

**QUESTION 4: Dental Screening**

Do inmates receive a routine dental screening during the medical intake? [circle one]

A. Yes

B. No

C. Don't know

**QUESTION 5: Mental Health Screening**

Does your jail system conduct the following mental health screenings during the medical intake?

| **INSTRUCTIONS:** Check all that apply (at least one response per row). | | | | | |
|---|---|---|---|---|---|
| Tests | Yes | | | No | Don't Know |
| | Self-reported by inmate | Validated clinical screening questionnaire | State/county mandated screening questionnaire | | |
| a) Mental health problems (excluding suicide risk) | ☐ | ☐ | ☐ | ☐ | ☐ |
| b) Suicide risk | ☐ | ☐ | ☐ | ☐ | ☐ |
| c) Traumatic brain injury | ☐ | ☐ | ☐ | ☐ | ☐ |
| d) Drug abuse/addiction problems | ☐ | ☐ | ☐ | ☐ | ☐ |

**QUESTION 6: Employee**

Who conducts the medical intake? [circle one]

A. correctional officer

B. civilian staff person

C. registered nurse (RN)

D. licensed practical nurse (LPN) or licensed vocational nurse (LVN)

E. medical practitioner (physician [MD/DO], physician assistant, or nurse practitioner)

F. other medical personnel (e.g., paramedic, EMT, nurses aid)

**QUESTION 7: Timing of medical intake**

How long after booking is the medical intake expected to occur? [write-in/circle]

within _____ A. minutes

B. hours

C. days

D. weeks

## PART 2: Non-Urgent Episodic Care (Sick Call)

**QUESTION 8: Triage**

When an inmate submits a request to be seen for a health issue, who triages the request? [circle one]

A. correctional officer (CO)

B. civilian staff person

C. registered nurse (RN)

D. licensed practical nurse (LPN) or licensed vocational nurse (LVN)

E. medical practitioner (physician [MD/DO], physician assistant, or nurse practitioner)

F. other medical personnel (e.g., paramedic, EMT, nurses aid)

G. requests are not triaged

H. patients do not submit requests, they just sign up for/ report to clinic

If you answered A-F, continue to **QUESTION 9**.

If you answered G or H, skip to **QUESTION 10**.

**QUESTION 9: Timing of Triage**

How long after an inmate submits a sick call request is triage expected to occur? [write-in/circle]

within _____ A. minutes

B. hours

C. days

D. weeks

**QUESTION 10: Episodic Care Initial Management**

Episodic care is an acute injury, illness, or new complaint. If an inmate submits a request for episodic care, who conducts the initial face-to-face evaluation? [circle one]

A. physician (MD/DO)

B. registered nurse (RN)

C. licensed practical nurse (LPN) or licensed vocational nurse (LVN)

D. other medical personnel (e.g., paramedic, EMT, nurses aid)

**QUESTION 11: Chronic Care Initial Management**

If an inmate submits a request for episodic care related to a chronic condition (e.g., diabetes, hypertension) who conducts the initial face-to-face medical evaluation? [circle one]

A. physician (MD/DO)

B. registered nurse (RN)

C. licensed practical nurse (LPN) or licensed vocational nurse (LVN)

D. other medical personnel (e.g., paramedic, EMT, nurses aid)

**QUESTION 12: Ongoing Chronic Care Management**

If an inmate has a chronic health problem that requires ongoing care (e.g., regular blood draws, physical exams, diet management), who conducts scheduled visits to manage stable chronic care conditions? [circle one]

A. physician (MD/DO)

B. registered nurse (RN)

C. licensed practical nurse (LPN) or licensed vocational nurse (LVN)

D. other medical personnel (e.g., paramedic, EMT, nurses aid)

# PART 3: Substance Abuse Treatment

## QUESTION 13: Types of Treatment

Does your jail provide the following substance abuse treatment services?

| **INSTRUCTIONS:** Check all that apply | Yes |
|---|---|
| a) Drug/alcohol education | ☐ |
| b) Self-help groups (AA, NA) | ☐ |
| c) Group counseling | ☐ |
| d) Individual counseling | ☐ |
| e) Medication assisted therapy (MAT; e.g., methadone, buprenorphine [Suboxone ®, Subutex ®], naltrexone [Vivitrol ®]) | ☐ |
| f) Detoxification services (with or without MAT) | ☐ |

## QUESTION 14: Medication Assisted Therapy

If an inmate has an opiate use disorder (e.g., addicted to heroin or prescription opiates) when they enter your facility, do you provide the following medication assisted therapies (MAT)?

| **INSTRUCTIONS:** Check all that apply (at least one response per row). | | | | | | |
|---|---|---|---|---|---|---|
| | Continue use if a prescription is verified | Initiate for detox only | Offer to initiate after any detox | Provide at discharge | Provide linkage to treatment at discharge | This is not available at all |
| For **pregnant** inmates only | | | | | | |
| Methadone | ☐ | ☐ | ☐ | ☐ | ☐ | ☐ |
| Buprenorphine | ☐ | ☐ | ☐ | ☐ | ☐ | ☐ |
| Naltrexone | ☐ | ☐ | ☐ | ☐ | ☐ | ☐ |
| For **all** inmates | | | | | | |
| Methadone | ☐ | ☐ | ☐ | ☐ | ☐ | ☐ |
| Buprenorphine | ☐ | ☐ | ☐ | ☐ | ☐ | ☐ |
| Naltrexone | ☐ | ☐ | ☐ | ☐ | ☐ | ☐ |

# PART 4: Medical Contracting, Medical Staffing, and Location of Services

**QUESTION 15: Medical Contracting**

Does your jail have a contract agreement (e.g., the health care providers are NOT employees of the jail) for the following health care services provided to inmates?

*For example, it is possible to have some mental health care services contracted to a public agency and some provided by employees of the jail. It is also possible that all of your dental services are contracted with a for-profit (private) company, and laboratory testing is not provided at your jail.*

**INSTRUCTIONS:** Check all that apply (at least one response per row).

| Health care services | **Contracted to public agency** (e.g., county hospital, public health department) | | **Contracted to university or university hospital** | | **Contracted to for-profit (private) company** | | **These services are provided by jail employees** | | **Other** | |
|---|---|---|---|---|---|---|---|---|---|---|
| | All contracted | Some contracted | All contracted | Some contracted | All contracted | Some contracted | All | Some | Don't know | Service Not Provided |
| a) Mental health | ☐ | ☐ | ☐ | ☐ | ☐ | ☐ | ☐ | ☐ | ☐ | ☐ |
| b) Laboratory testing (other than finger stick, drug urinalysis, pregnancy test, etc.) | ☐ | ☐ | ☐ | ☐ | ☐ | ☐ | ☐ | ☐ | ☐ | ☐ |
| c) Radiation technician services (e.g., x-ray) | ☐ | ☐ | ☐ | ☐ | ☐ | ☐ | ☐ | ☐ | ☐ | ☐ |
| d) Dental | ☐ | ☐ | ☐ | ☐ | ☐ | ☐ | ☐ | ☐ | ☐ | ☐ |
| e) Medical (excluding all of the above) | ☐ | ☐ | ☐ | ☐ | ☐ | ☐ | ☐ | ☐ | ☐ | ☐ |

**QUESTION 16: Medical Staffing**

On June 30, 2017 (mid-year 2017), in total, how many full-time equivalent (FTE) personnel (employed or contracted) did you have working at the jail?

*For example, an x-ray technician from the county hospital that works in your jail 2 hours per week would be 0.05 FTE and would count as "q. other medical staff."*

| **INSTRUCTIONS:** Write-in response | | |
|---|---|---|
| Employee type | Number FTE | If estimate, check this box |
| **Mental health**<br>a) Psychiatrists (MD, DO) | | ☐ |
| b) Psychiatric physician assistants or nurse practitioner | | ☐ |
| c) Psychiatric nurses (PMHCNS) | | ☐ |
| d) Clinical psychologists (PhD, PsyD, MS) | | ☐ |
| e) Clinical social workers (LCSW) or other masters-level counselor | | ☐ |
| f) Other mental health staff | | ☐ |
| **Pharmaceutical**<br>g) Pharmacists (DPh, RPh) | | ☐ |
| h) Other pharmaceutical staff | | ☐ |
| **Dental**<br>i) Dentists (DDS) | | ☐ |
| j) Dental hygienists/assistants | | ☐ |
| **Medical only**<br>k) Physician assistants (PA) or Nurse practitioner (NP) | | ☐ |
| m) Registered nurse (RN) | | ☐ |
| n) Licensed practical nurse (LPN), licensed vocational nurse (LVN) | | ☐ |
| o) Physicians (MD/DO) | | ☐ |
| p) Paramedic/Emergency Medical Technician (EMT) | | ☐ |
| q) Other medical staff (e.g., CNA, etc.) | | ☐ |

**QUESTION 17: Location of Services Provided**

Does your jail system provide the following health care services, either on-site or off-site?

*For example, your jail may provide dental services both on-site (at the jail) and off-site (in the community). In this case, you would check "yes" for both columns.*

| **INSTRUCTIONS:** Check all that apply (at least one response per row). | | | | | |
|---|---|---|---|---|---|
| Services | On-site (at jail) | | Off-Site (in community) | | Service Not Provided |
| | Yes | Don't Know | Yes | Don't Know | |
| a) Inpatient mental health (overnight) | ☐ | ☐ | ☐ | ☐ | ☐ |
| b) Outpatient mental health | ☐ | ☐ | ☐ | ☐ | ☐ |
| c) Inpatient medical health care (overnight) | ☐ | ☐ | ☐ | ☐ | ☐ |
| d) Outpatient medical health care (i.e., infirmary or sick call) | ☐ | ☐ | ☐ | ☐ | ☐ |
| e) Dialysis (i.e., for kidney disease) | ☐ | ☐ | ☐ | ☐ | ☐ |
| f) Chronic care other than dialysis (e.g., wound care, pain management) | ☐ | ☐ | ☐ | ☐ | ☐ |
| g) Dental care | ☐ | ☐ | ☐ | ☐ | ☐ |
| h) 24-hour physician or nurse coverage (i.e., "after hours" coverage) | ☐ | ☐ | ☐ | ☐ | ☐ |
| i) Emergency department care | ☐ | ☐ | ☐ | ☐ | ☐ |
| j) Inpatient surgeries/ operation (overnight) | ☐ | ☐ | ☐ | ☐ | ☐ |
| k) Outpatient surgeries/ operations | ☐ | ☐ | ☐ | ☐ | ☐ |
| l) Long-term nursing home care (e.g., geriatric, assisted living, etc.) | ☐ | ☐ | ☐ | ☐ | ☐ |
| m) Hospice care | ☐ | ☐ | ☐ | ☐ | ☐ |

## PART 5: Miscellaneous

**QUESTION 18: Accreditation**

Is your jail currently accredited by any of the following organizations? [circle all that apply]

A. American Correctional Association (ACA)

B. National Commission on Correctional Health Care (NCCHC)

C. State or county level agency

D. Other, please specify: ________________

**QUESTION 19: HIV Medication 1**

Pre-exposure prophylaxis (PrEP) is a new medication regimen that can *prevent* HIV for people that are at high risk for getting HIV such as men who have sex with men and people who inject drugs. Does your jail administer PrEP to inmates who request it? [circle one]

A. Yes

B. No

C. Don’t know

**QUESTION 20: HIV Medication 2**

Does your jail provide a referral to PrEP during discharge planning? [circle one]

A. Yes

B. No

C. Don’t know

**QUESTION 21: Telemedicine**

In the past year, has your jail used telemedicine for any of the following services?

| **INSTRUCTIONS:** Check all that apply | Yes |
|---|---|
| a) Primary medical care | ☐ |
| b) Specialty medical care | ☐ |
| c) Mental health/ psychiatric care | ☐ |
| d) Any other health care (e.g., dental) | ☐ |

**QUESTION 22: Challenges**

What major challenges/issues is your jail system currently facing in regards to the delivery of health care? [write-in response]

**QUESTION 23: Medical Intake Forms**

Would you be willing to share a blank copy of your medical intake forms with the UM Jail Study research team? [circle one]

A. Yes

B. No

C. Maybe

If you are able, please include a copy of your medical intake forms or email seperately to jailstudy@miami.edu.

**THANK YOU FOR YOUR PARTICIPATION!**
